# Idiopathic Intracranial Hypertension in a Malaysian Preschooler

**DOI:** 10.7759/cureus.19207

**Published:** 2021-11-02

**Authors:** Siti Farhah 'Adilah Basiron, Ee Ling Tan, Tun Wang Ch'ng, Othmaliza Othman

**Affiliations:** 1 Ophthalmology, Universiti Kebangsaan Malaysia Medical Centre, Kuala Lumpur, MYS; 2 Ophthalmology, Hospital Raja Permaisuri Bainun, Perak, MYS

**Keywords:** false localizing sign, pediatric, pseudo tumor cerebri, papilledema, idiopathic intracranial hypertension (iih)

## Abstract

A four-year-old girl presented with a three-day history of squint and irritability. Examination showed reduced visual acuity in both eyes, the presence of a false localizing sign, and bilateral optic disc swelling. On investigation, her blood laboratory workup was within the normal range. Imaging of the brain showed no evidence of a space-occupying lesion or cerebral venous sinus thrombosis. The lumbar puncture opening pressure was 27cmH_2_0 and the cerebrospinal fluid workup was normal. The diagnosis of idiopathic intracranial hypertension (IIH) was made based on the diagnostic criteria for pseudotumor cerebri syndrome. She was successfully treated with acetazolamide with resolved symptoms and signs. This highlights the possibility of IIH presenting with inconspicuous symptoms in preschool children, which needs a high index suspicion by clinicians. Hence, solving the challenges in the workup, especially in children, is very crucial.

## Introduction

Discerned by an elevation of intracranial pressure and normal composition of cerebrospinal fluid with the absence of an intracranial space-occupying lesion, idiopathic intracranial hypertension (IIH) is a disorder without any primary causes [1]. The incidence of idiopathic intracranial hypertension in the general population is 0.03 to 2.36 per 100,000 per year [2]. There is a strong correlation between adult obese women and IIH, although no causative relationship has been demonstrated [3]. It rarely occurs in the pediatric population, especially at preschool age. We report a rare case of idiopathic intracranial hypertension in a four-year-old girl. This article was previously presented as a poster at the Asia-Pacific Association of Cataract and Refractive Surgeons and the Singapore National Eye Centre (APACRS-SNEC) 30th Anniversary virtual meeting held on July 30th and 31st, 2021.

## Case presentation

A four-year-old Malaysian girl presented with a three-day history of squint, whereby her right eye deviated inward. The squint was of sudden onset, persistent throughout the day, painless, and associated with emotional changes. The parents noted that she was constantly in an irritable mood without any apparent explanations or causes since the onset of the squint.

The child was otherwise well, with no history of worsening vision, diplopia, vomiting, headache, fever, tinnitus, neck stiffness, or seizures. She had no history of medical illness prior to the presentation.

She appeared fretful but was consolable enough to proceed with the examination. Her weight was 12.65kg with a height of 96cm. Her calculated BMI was 13.73kg/m2, placing her BMI-for-age at the fifth percentile for girls aged four years. This child falls under the healthy weight category according to the CDC growth chart for children and teens aged two through 19 years. Her visual acuity was 6/24 in both eyes. There was no relative afferent pupillary defect (RAPD) detected. Extraocular muscle examination showed lateral restriction (minus three) of right eye movement, which is consistent with right sixth cranial nerve palsy. However, other neurological examination findings were normal.

**Figure 1:**
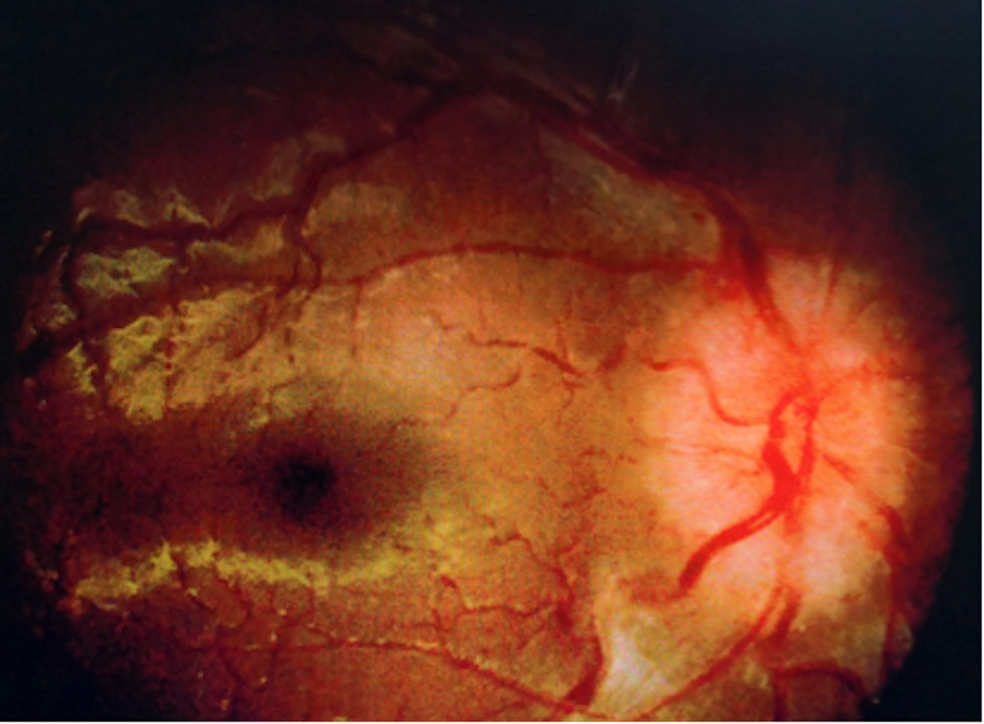
Fundus photo of oculus dexter (OD) shows optic disc swelling at presentation

**Figure 2:**
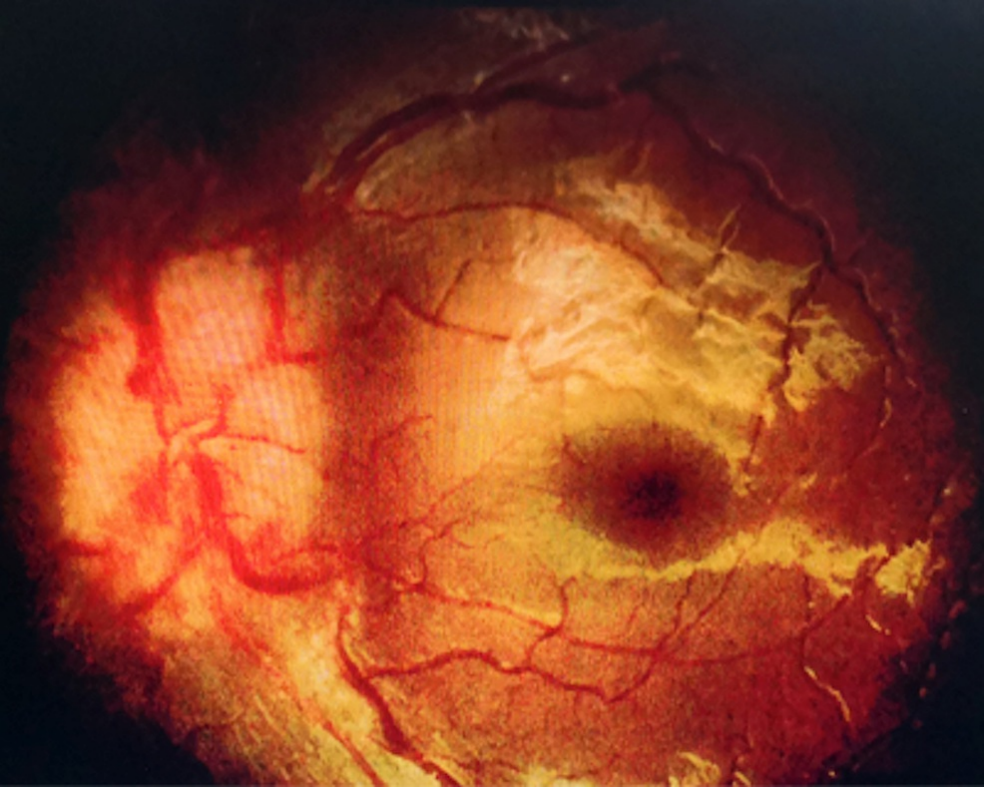
Fundus photo of oculus sinister (OS) shows optic disc swelling at presentation

Slit-lamp examination of both eyes revealed no anterior segment abnormalities. The fundus examination showed bilateral papilledema grade 2 (Frisen Scale). Both optic discs showed obscuration and elevation of disc borders with a complete peripapillary halo (Figure 1 and Figure 2). The retina was flat and the macula was normal with a good foveal reflex. There was no evidence of inflammation such as retinitis, choroiditis, vasculitis, or vitritis.

Blood laboratory workup was normal for full blood count, renal profile, liver function test, calcium, magnesium, and phosphate. The results indicate that there was no evidence of anemia or malnutrition. A lumbar puncture was performed in the lateral decubitus position under sedation. The opening pressure was 27cmH20. The cerebrospinal fluid (CSF) biochemistry was within normal range and there was no oligoclonal band seen in CSF electrophoresis, ruling out infection and demyelinating disease.

There was no evidence of a space-occupying lesion, cerebral venous sinus thrombosis, hydrocephalus, or demyelinating disease on MRI or magnetic resonance venography (MRV) of the brain. 

**Figure 3:**
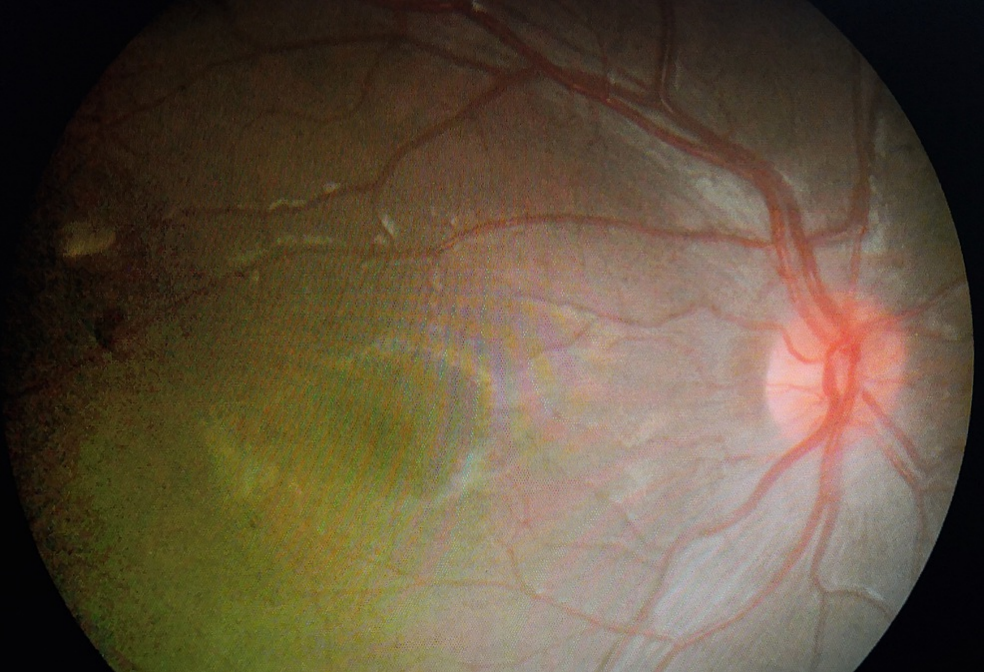
Optic disc of oculus dexter (OD) at six weeks follow-up showing improvement of optic disc swelling

**Figure 4:**
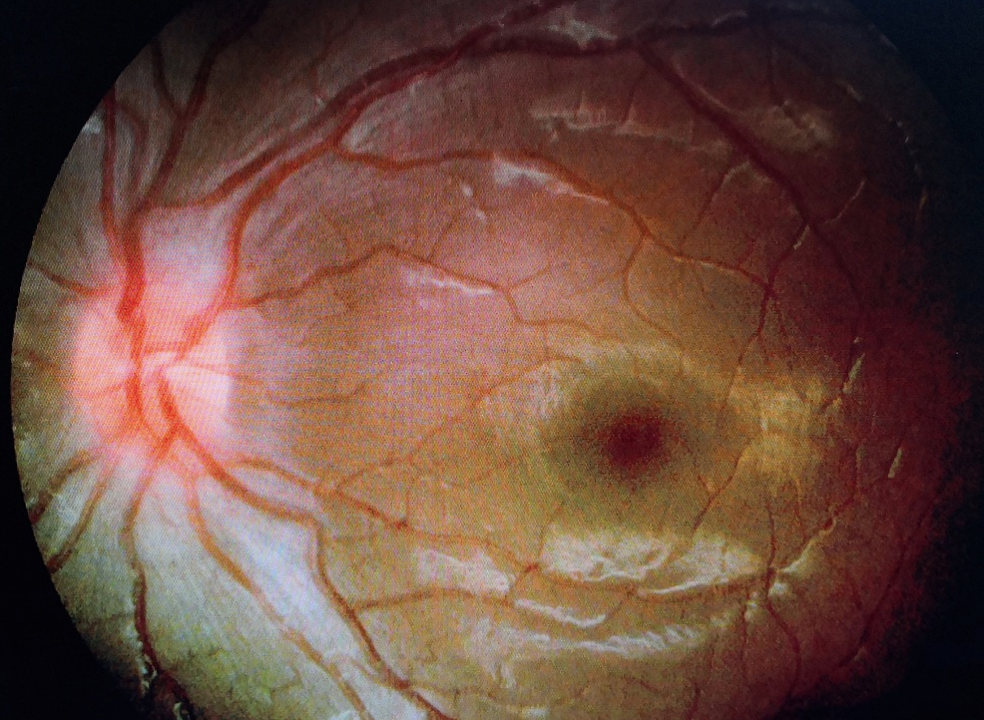
Optic disc of oculus sinister (OS) at six weeks follow-up showing improvement of optic disc swelling

The patient was diagnosed with idiopathic intracranial hypertension based on the modified Dandy criteria. She was co-managed by the pediatric team and was prescribed acetazolamide 10mg/kg/8 hours with regular monitoring of her renal profile. Six weeks following initiation of treatment, her vision improved to right eye 6/12 and left eye 6/15. Her papilledema was improving (Figure 3 and Figure 4) and the sixth cranial nerve palsy resolved. Unfortunately, the patient was lost to follow-up thereafter and remained uncontactable. 

## Discussion

Idiopathic intracranial hypertension (IIH), also known as pseudotumor cerebri, commonly occurs in the adult population. The incidence of IIH in the pediatric population is rare, especially at preschool age. Globally, the incidence of IIH in children varies in different locations. Recent studies in Britain and the United States estimate the annual pediatric incidence of IIH to be 0.71 and 0.63 per 100,000, respectively [4]. To date, a limited number of pediatric IIH have been reported in Asian countries.

Pediatric IIH can be divided into prepubescent and pubescent groups. Female sexual predilection is seen among the pubertal age group of IIH [5]. Various studies have identified obesity as a risk factor for IIH in the pubertal age group [5,6]. By contrast, in the prepubertal age group, there is no gender predominance and obesity is not a risk factor for IIH [7,8].

Pediatric IIH may be asymptomatic or present with symptoms of increased intracranial pressure. Asymptomatic patients usually have milder IIH and are detected through incidental findings of papilledema during eye assessment [9]. The common symptoms of increased intracranial pressure are headaches, diplopia, blurring of vision, vomiting, tinnitus, and neck stiffness [6]. This patient, however, presented with vague symptoms of irritability and squint, similar to those reported in previous studies [10].

Despite an indistinct presentation history, a detailed physical examination revealed classical findings of IIH such as right sixth cranial nerve palsy and papilledema. Subsequent workup was done urgently and the patient was also co-managed with the pediatric team to determine the underlying cause.

Through the process of elimination and fulfillment of diagnostic criteria for pseudotumor cerebri syndrome proposed by Friedman et al., this patient was diagnosed with probable pediatric IIH [1]. The required criteria for the diagnosis of pseudotumor cerebri syndrome comprise the following: A) papilledema; B) normal neurological examination except for cranial nerve abnormalities; C) neuroimaging i.e., normal brain parenchyma without evidence of hydrocephalus, mass or structural lesion, and no abnormal meningeal enhancement on MRI with and without gadolinium for typical patients (female and obese), MRI with and without gadolinium and MRV for others, and if MRI is unavailable or contraindicated, contrast-enhanced CT may be used; D) normal CSF composition and E) elevated lumbar puncture opening pressure [≥25 cmH20 in adults and ≥28cmH20 in children (25cmH20 if the child is not sedated and not obese)] in a properly performed lumbar puncture. With this patient, the diagnosis of IIH was probable as she fulfilled all the criteria from A to D, but the measured lumbar puncture opening pressure was lower than specified for a definite diagnosis.

Randomized clinical trials are limited in pediatric IIH due to its rare incidence. Thus, the treatment for pediatric IIH is largely based on evidence procured from the adult population [11]. The mainstay of treatment is lowering intracranial pressure (ICP) to preserve vision and control symptoms. Fortunately, this patient responded well to the carbonic anhydrase inhibitor, acetazolamide. Thus, she did not require any surgical intervention to reduce the ICP. Most children with mild to moderate visual field abnormalities will resolve with early diagnosis and intervention [12].

## Conclusions

This case report highlights that vague symptoms of IIH in preschool children pose a challenge in diagnosing the condition. Prompt diagnosis and treatment are required to prevent future complications that decrease patients’ quality of life. 
